# On modeling the correlates of conspiracy thinking

**DOI:** 10.1038/s41598-023-34391-6

**Published:** 2023-05-23

**Authors:** Adam M. Enders, Amanda Diekman, Casey Klofstad, Manohar Murthi, Daniel Verdear, Stefan Wuchty, Joseph Uscinski

**Affiliations:** 1 Department of Political Science, University of Louisville, Louisville, KY 40292 USA; 2 Department of Psychology, Indiana University, Bloomington, IN 47405 USA; 3 Department of Political Science, University of Miami, Coral Gables, FL 33146 USA; 4 Department of Electrical and Computer Engineering, University of Miami, Coral Gables, FL 33146 USA; 5 Department of Computer Science, University of Miami, Coral Gables, FL 33146 USA; 6 Department of Biology, University of Miami, Coral Gables, FL 33146 USA; 7 Institute of Data Science and Computing, University of Miami, Coral Gables, FL 33146 USA; 8 Sylvester Comprehensive Cancer Center, University of Miami, Miami, FL 33136 USA

**Keywords:** Human behaviour, Psychology and behaviour

## Abstract

While a robust literature on the psychology of conspiracy theories has identified dozens of characteristics correlated with conspiracy theory beliefs, much less attention has been paid to understanding the generalized predisposition towards interpreting events and circumstances as the product of supposed conspiracies. Using a unique national survey of 2015 U.S. adults from October 2020, we investigate the relationship between this predisposition—conspiracy thinking—and 34 different psychological, political, and social correlates. Using conditional inference tree modeling—a machine learning-based approach designed to facilitate prediction using a flexible modeling methodology—we identify the characteristics that are most useful for orienting individuals along the conspiracy thinking continuum, including (but not limited to): anomie, Manicheanism, support for political violence, a tendency to share false information online, populism, narcissism, and psychopathy. Altogether, psychological characteristics are much more useful in predicting conspiracy thinking than are political and social characteristics, though even our robust set of correlates only partially accounts for variance in conspiracy thinking.

## Introduction

Conspiracy theories^1–4^ are typically defined as accounts of events or circumstances that “assert that some small and hidden group has through special means, powers, or manipulations brought about visible and evil effects of whose true cause most people are unaware”^5^. A robust literature has identified dozens of psychological, political, and social characteristics that correlate with beliefs in conspiracy theories^6^. For example, beliefs in conspiracy theories doubting the veracity of the Holocaust or of the mass shooting in Sandy Hook, CT are correlated with dark triad personality traits, support for the use of political violence, and a willingness to knowingly share false information online^7^. Conspiracy theory beliefs about election fraud are correlated with anomie and—depending on the groups and figures involved in the supposed fraud—partisan and ideological orientations^8^. Demographic traits such as gender^9^, educational attainment^10^, and race^11^ are also related to beliefs in some conspiracy theories, as are political attitudes, including support for partisan political figures^12^, distrust of government^13^, populist attitudes^14^, and Manichean thinking^15^.

This literature—of which only a small fraction can be cited here—produces two conclusions: (1) there are a great many correlates of specific conspiracy theory beliefs and (2) the correlates can differ considerably depending on which specific conspiracy theory belief is being probed^16^. To illustrate the second conclusion, consider the conspiracy theory that former U.S. president Barack Obama faked his birth certificate. Beliefs in this theory are highly correlated with partisan and ideological identities—very few Democrats or liberals believe this, though some Republicans and conservatives do^17^. Whereas these “birther” beliefs are correlated with partisan and ideological orientations, beliefs in many other conspiracy theories that do not involve parties, partisan political figures, or ideological principles are unrelated to political orientations^7^. In other words, the factors that explain beliefs in one conspiracy theory, such as the “birther” theory, might not explain beliefs in other conspiracy theories; likewise, beliefs in specific conspiracy theories cannot be used to make inferences about a tendency toward conspiratorial thinking, more generally.

In reaction to this, researchers have theorized about and developed empirical measures of a general predisposition to interpret events and circumstances as the product of conspiracies^18–23^. This predisposition is usually referred to as conspiracy thinking (the term we use), conspiracy mentality, or conspiracy ideation. By both recognizing a psychological tendency to view the world through a lens of conspiracy and devising strategies to measure this tendency that are not burdened by the idiosyncratic details of specific conspiracy theories, scholars have produced a more generalizable way to study cosnpiracism^16^.

Despite the utility of the conspiracy thinking construct, researchers have been slow to understand the correlates of the generalized disposition compared to the correlates of specific conspiracy theory beliefs. Conspiracy thinking is one of very few—if not the only—correlate of specific conspiracy theory beliefs that appears to be related to conspiracy theory beliefs regardless of the details of specific theory in question^7^. While numerous studies have taken this important step in validating the conspiracy thinking construct^9,12,20,24–26^, it does not illuminate much about the nature of conspiracy thinking. And, even though recent work explores—and disagrees on—the relationship between conspiracy thinking and partisan and ideological orientations^13,20,27^, fairly little is known about the psychological, political, and social characteristics of those disposed toward conspiracy thinking beyond this emerging work. Moreover, what literature does examine the correlates of conspiracy thinking has proceeded in a fairly piecemeal fashion, oftentimes only examining a single new potential correlate at a time and only controlling for a limited number of potential confounders. This procession, while natural, leaves the scholarly community unable to answer basic questions about which political, psychological, and social characteristics seem to be most consistently and strongly related to conspiracy thinking, and under what conditions. In short, more must be done to understand who is most (least) prone to believing conspiracy theories in general.

In this study, our goal is twofold: (1) examine a large set of potential correlates that span the psychological, political, and social domains, and (2) decipher how well these correlates explain conspiracy thinking when they are considered in tandem. As the list of previously-identified correlates of either beliefs in specific conspiracy theories or conspiracy thinking, specifically, is too long to allow for a complete, exhaustive analysis using a single survey, we used the literature review conducted by Douglas et al.^6^ as well as recent literature emphasizing the importance of political predictors and “dark” personality traits in the aftermath of the 2020 U.S. presidential election and January 6th riot to aid in a determination of which correlates to focus on. We list and define each of these correlates in Table 1, where we also supply citations to previous work identifying a relationship between the construct in question and conspiracism.

**Table 1:** Constructs that are related to conspiracism, with definitions of constructs. Citations regard the measurement of constructs and previously-identified relationships between the constructs and conspiracism.

Constructs	Definition/operationalization
Anomie	A tendency to believe that social conditions and institutions are irreparably crumbling; typically a marker for social alienation^58,59^
Argumentative	A tendency to argue with others, especially when beliefs clash^60^
Dogmatism	A tendency to stubbornly and narrow-mindedly cling to one’s beliefs, while disregarding or derogating the beliefs of others, regardless of evidence^60,61^
Machiavellianism	An anti-social personality trait characterized by willingness to manipulate others toward the end of gaining power^62–65^
Narcissism	An anti-social personality trait characterized by an exaggerated sense of self-importance and need for admiration by others^62–65^
Psychopathy	An anti-social personality trait characterized by a lack of empathy and remorse, and egotism^62–65^
Distrust of government	Typically operationalized as attitudinal distrust of government^58^
Distrust of police	Typically operationalized as attitudinal distrust of police^58^
Interest in politics	Typically operationalized as the level of interest in or time spent following political news or campaigns^12,15^
Manicheanism	A tendency to view life as a constant struggle between good and evil^15,66^
National narcissism	A tendency to hold an inflated, exaggerated view of one’s nation^67,68^
Populism	A tendency to view politics as a struggle between the inherently good “people” and the corrupt, evil “elite”^14,56,63,69–71^
Support political violence	A tendency to believe that political violence is justified, at least under certain conditions^7,63^
Partisanship	In the U.S. context, operationalized as Republican (right), Democratic (left), or Independent (center) identification and strength thereof^72,73^
Ideology	In the U.S. context, operationalized as conservative (right), liberal (left), or moderate (center) identification and strength thereof^13,74,75^
Trump support	Typically operationalized using vote choice or feelings toward Trump^12,64^
Social media usage	Typically operationalized as the frequency of time spent on various platforms^33,76^
Share false info. online	A willingness to share online information one knows to be false^7^
Age	Typically operationalized using age in years^24,58^
Education	Typically operationalized using years of education or completed degrees^10,30,77^
Gender	Past work tends to focus on discrete gender categories (e.g., man, woman)^9^
Household income	Typically operationalized using income brackets, sometimes class^30^
Religiosity	Typically operationalized using frequency of attendance of religious services^78,79^
Race and ethnicity	Past work tends to focus on White, Black, and Hispanic individuals^56,80^

In addition to examining the correlations between these characteristics and conspiracy thinking, we utilize conditional inference trees—a machine learning-based approach designed to facilitate prediction using a flexible modeling methodology^28^—to determine which correlates exhibit the most utility in explaining variance in conspiracy thinking, allowing for non-linear and interactive relationships between the correlates. Our results shed light on which specific correlates and broader classes of correlates are most useful in predicting conspiracy thinking. Finally, we examine the typical profile—across the correlates we examine—of individuals exhibiting high and low levels of conspiracy thinking. Our findings have several implications for the study of conspiracy thinking and for societal efforts to address conspiracy theory beliefs.

## Materials and methods

### Survey

To examine the predictors of conspiracy thinking, we fielded a national survey on U.S. adults in October 2020, in partnership with Qualtrics. The sample is reflective of the U.S. adult population in terms of age, gender, educational attainment, income, and race and ethnicity, although the sample is quota-based rather than purely random. We took several steps to ensure the quality of our data—each of which were determined before data collection and were executed on our behalf by Qualtrics before delivering the final dataset. First, respondents who did not pass three attention checks were automatically removed from the dataset. Respondents who spent less time on the survey than one standard deviation below the median completion time were also removed. Both of these decisions were made before data was collected by Qualtrics and delivered to us. The final sample is composed of 2015 U.S. adults. The supplementary information contains information about the precise sociodemographic composition of the sample.

The survey protocol was approved by University of Miami’s institutional review board (Protocol #20201154). Survey respondents provided informed consent via computer screen and could leave the survey at any time. This research was performed in accordance with all relevant guidelines and regulations, and with the Declaration of Helsinki. All data analyzed during this study are included in the supplementary information files (“Dataset 1”). All data and replication code is available in the Open Science Framework.

Several measures of a predisposition towards believing conspiracy theories have been developed and validated across fields^18,19,21–23,29^. We use Uscinski and Parent’s (2014) American Conspiracy Thinking Scale (ACTS)^30^, which is an additive index of responses—on a five-point “strongly disagree” (1) to “strongly agree” (5) scale—to the following four items:

1. Much of our lives are being controlled by plots hatched in secret places.
2. Even though we live in a democracy, a few people will always run things anyway.
3. The people who really “run” the country are not known to the voters.
4. Big events like wars, the current recession, and the outcomes of elections are controlled by small groups of people who are working in secret against the rest of us.

**Figure 1:**
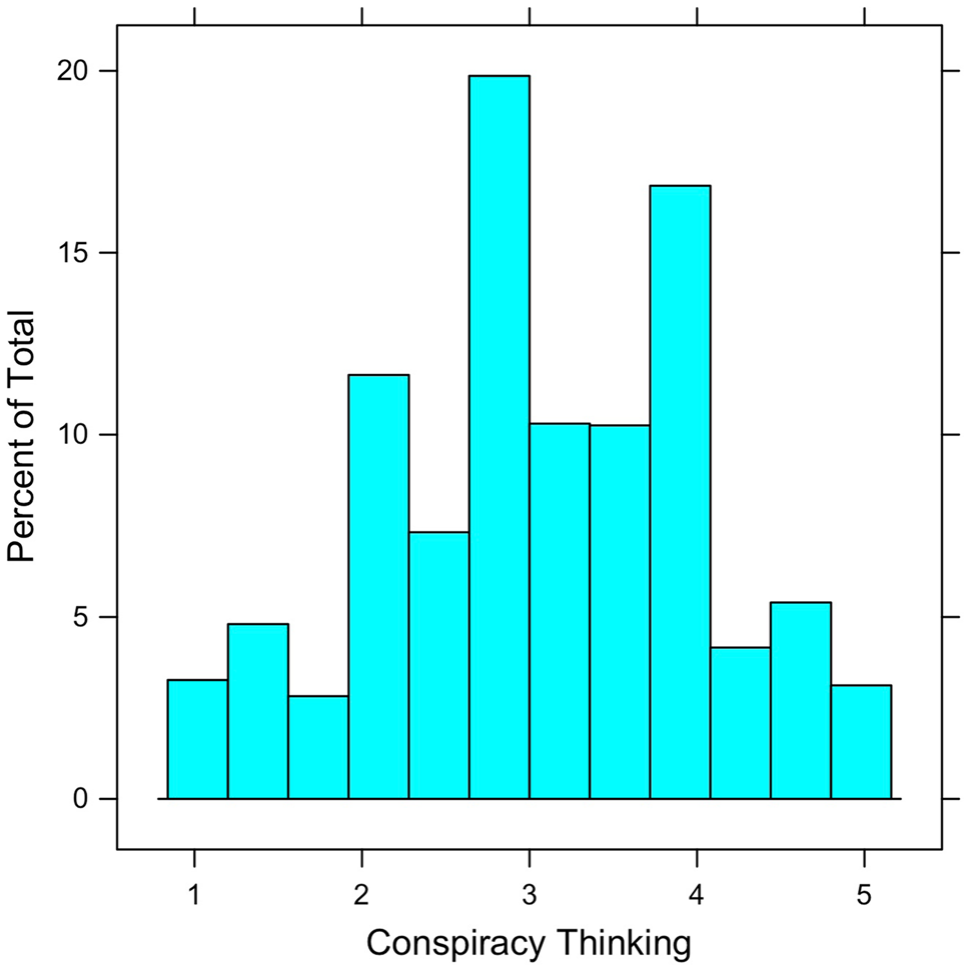
Distribution of American Conspiracy Thinking Scale (ACTS). The mean is 3.09 on a scale ranging from 1 (low conspiracy thinking) to 5 (high conspiracy thinking).

This scale (Range = 1–5, *M* = 3.09, *SD* = 0.95) is statistically reliable (α = 0.84), unidimensional, and has been validated using U.S.^9,24,26,31^ and international data^32^. Figure 1 displays the distribution of the ACTS, which is fairly symmetric, save for a minor negative skew. Extreme levels of conspiracy thinking may be uncommon, but most Americans exhibit middling levels.

**Figure 2:**
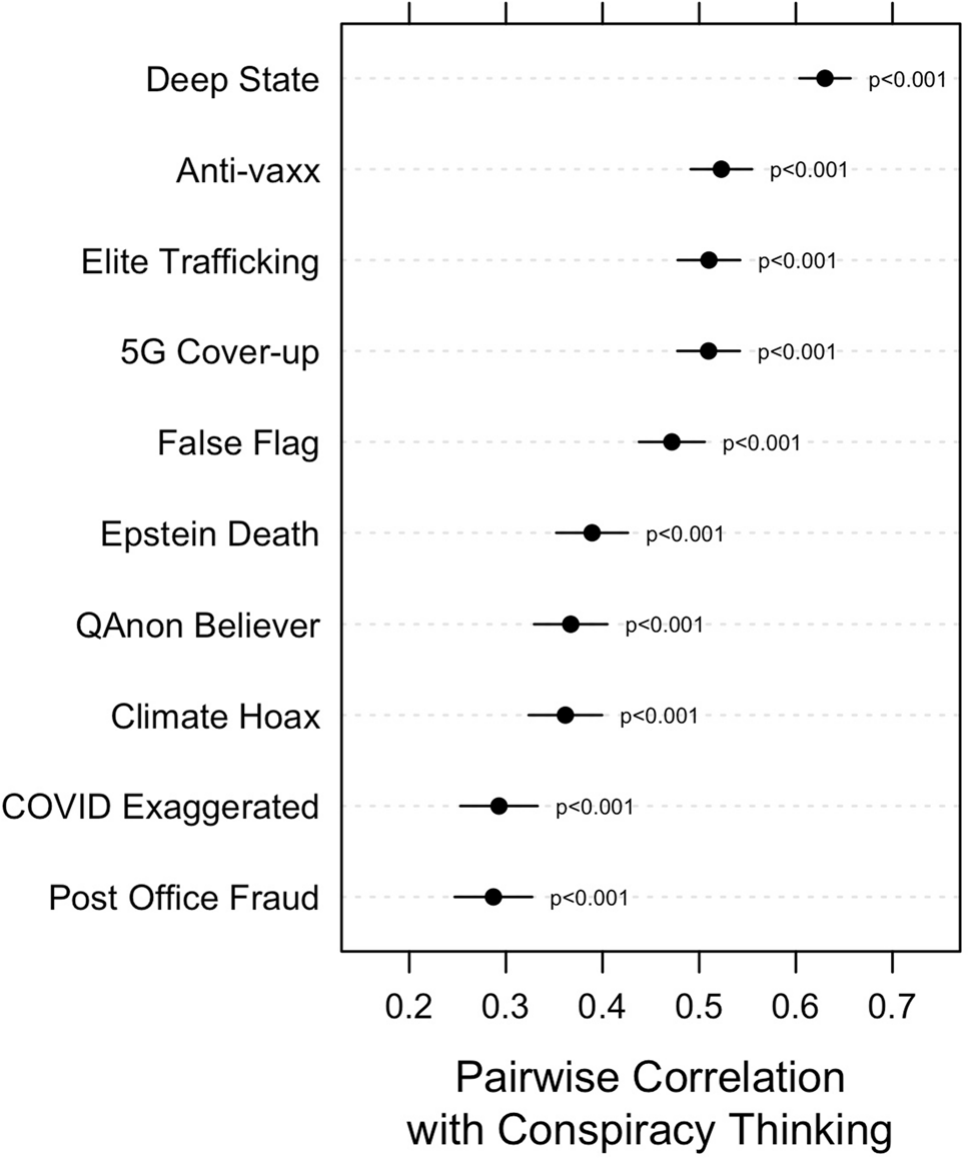
Pearson correlations between ACTS and beliefs in 10 specific conspiracy theories. Horizontal bars represent 95% confidence intervals. Bonferroni corrections for multiple comparisons were to compute *p*-values.

We highlight the criterion validity of the ACTS in Fig. 2, which displays the pairwise correlations between the ACTS and beliefs in 10 specific conspiracy theories (see the supplemental information for precise question wording and the percentage of respondents believing each conspiracy theory). In each case, correlations are positive and statistically significant (*p* < 0.001 in each case), ranging in magnitude from 0.29 to 0.63. Even though measures of conspiracy thinking, like the ACTS, are not a direct substitute for beliefs in all specific conspiracy theories, they tend to be positively related to specific conspiracy theory beliefs, making them useful indicators of one’s propensity to interpret events and circumstances in conspiratorial terms and to believe specific conspiracy theories. Thus, measures of conspiracy thinking are worthy of more careful investigation than the extant literature has afforded them.

We examine the relationship between conspiracy thinking and 34 different psychological, political, and social characteristics. While no list of potential correlates is exhaustive, especially given the rapidly expanding research into conspiracy theory beliefs, our list offers broad coverage of the types of factors that (1) exhibit the strongest or most consistent relationship with specific conspiracy theories in past work (psychological and non-partisan/ideological political orientations)^6,8^, (2) have been hypothesized to relate to conspiracy thinking or conspiracy theory beliefs, though evidence is currently weak or mixed (e.g., social media use, partisan and ideological orientations)^27,33^, or (3) have yet to be sufficiently analyzed (e.g., sociodemographic characteristics). We list each of these potential correlates in Table 2, along with descriptive statistics, in sections (from top to bottom): psychological traits, non-partisan/ideological political orientations, partisan/ideological political orientations, online behavior and social media use, and sociodemographic characteristics. See the supplemental information for exact question wording and scale construction.

**Table 2:** Correlates of conspiracy thinking and descriptive statistics, including the range, mean, standard deviation, as well as Cronbach’s alpha and the number of items for multiple-item scales.

Variable	Range	Mean	Std. dev.	# Items	Alpha
Psychological					
Anomie	1–5	3.56	0.78	3	0.62
Argumentative	1–5	2.86	0.98	3	0.72
Dogmatism	1–5	3.13	0.83	3	0.60
Machiavellianism	1–5	2.11	0.91	4	0.84
Narcissism	1–5	2.35	0.98	4	0.88
Psychopathy	1–5	2.13	0.85	4	0.81
Political, non-partisan/ideological					
Distrust government	1–5	3.36	1.08		
Distrust police	1–5	2.58	1.19		
Interest in politics	1–4	4.12	0.99		
Manicheanism	1–5	3.27	1.15		
National narcissism	1–5	3.05	0.93	3	0.75
Populism	1–5	3.84	0.63	9	0.84
Support political violence	1–5	2.20	1.28		
Political, partisan/ideological					
Biden support	0–100	49.51	37.80		
Ideology (conservative)	1–7	4.03	1.74		
Ideological extremity	1–4	2.30	1.14		
Partisanship (republican)	1–7	3.83	2.23		
Partisan extremity	1–4	2.94	1.11		
Trump support	0–100	40.45	40.44		
Social media activity					
Facebook usage	1–5	3.78	1.57		
Twitter usage	1–5	2.43	1.69		
Instagram usage	1–5	2.88	1.78		
Reddit usage	1–5	1.82	1.30		
YouTube usage	1–5	3.73	1.44		
4chan/8chan usage	1–5	1.22	0.72		
Share false info. online	1–5	1.81	1.05		
Sociodemographic characteristics					
Age	18–90	44.67	17.96		
Education	1–6	3.69	1.40		
Gender (0 = Male, 1 = Female)	0, 1	0.51	0.50		
Household income	1–7	3.22	1.71		
Religiosity	1–5	2.25	1.25		
Black (0 = not, 1 = Black)	0, 1	0.14	0.34		
Hispanic (0 = not, 1 = Hispanic)	0, 1	0.18	0.38		
White (0 = not, 1 = White)	0, 1	0.68	0.47		

### Methods

We begin our investigation by examining correlations between conspiracy thinking and each of the 34 predictors listed above; all *p*-values are corrected for multiple comparisons using the Bonferroni procedure. This analysis is designed to showcase the relative strength and direction of the bivariate relationship between conspiracy thinking and many of the correlates that past work has identified.

Next, we determine which correlates exhibit the most predictive power in explaining variance in our measure of conspiracy thinking. One method for answering such a question is linear regression. We could simply regress the ACTS on the 34 correlates we consider and determine the direction, strength, and statistical significance of the resultant coefficients—we have done this, placing the results in the supplementary information. While straightforward, such a procedure has notable drawbacks. First, many of the correlates we consider are not only strongly related with conspiracy thinking, but with each other, introducing the (potential) problem of multicollinearity; see the supplementary information for correlations between predictors. Second, the correlates we identified may be non-linearly related to conspiracy thinking, or there could be important interactions between correlates (i.e., conditional relationships). While we could check each of these possibilities one at a time, or in batches, the advantage of the linear regression model—its simplicity—would quickly be lost.

Instead, we utilize a conditional inference tree model (sometimes called “classification and regression tree,” or CART), which is part of the broader family of classification-focused machine learning methods, to determine which predictors—when considered together—are most predictive of conspiracy thinking. Conditional inference trees and related methods are preferable to simpler linear models, for example, when the functional form of relationships is unknown, when there may be many interactions between predictor variables, and when multicollinearity is a potential issue (which is, itself, more likely when there is a large number of predictors)^28^. The goal of conditional inference tree models is to correctly classify values of a dependent variable (conspiracy thinking, in this case), allowing for complex non-linear and interactive relationships between the predictor variables. In this sense, the primary focus on this methodology is prediction, though we explicitly note that our cross-sectional data does not allow us to make inferences about causality.

**Figure 3:**
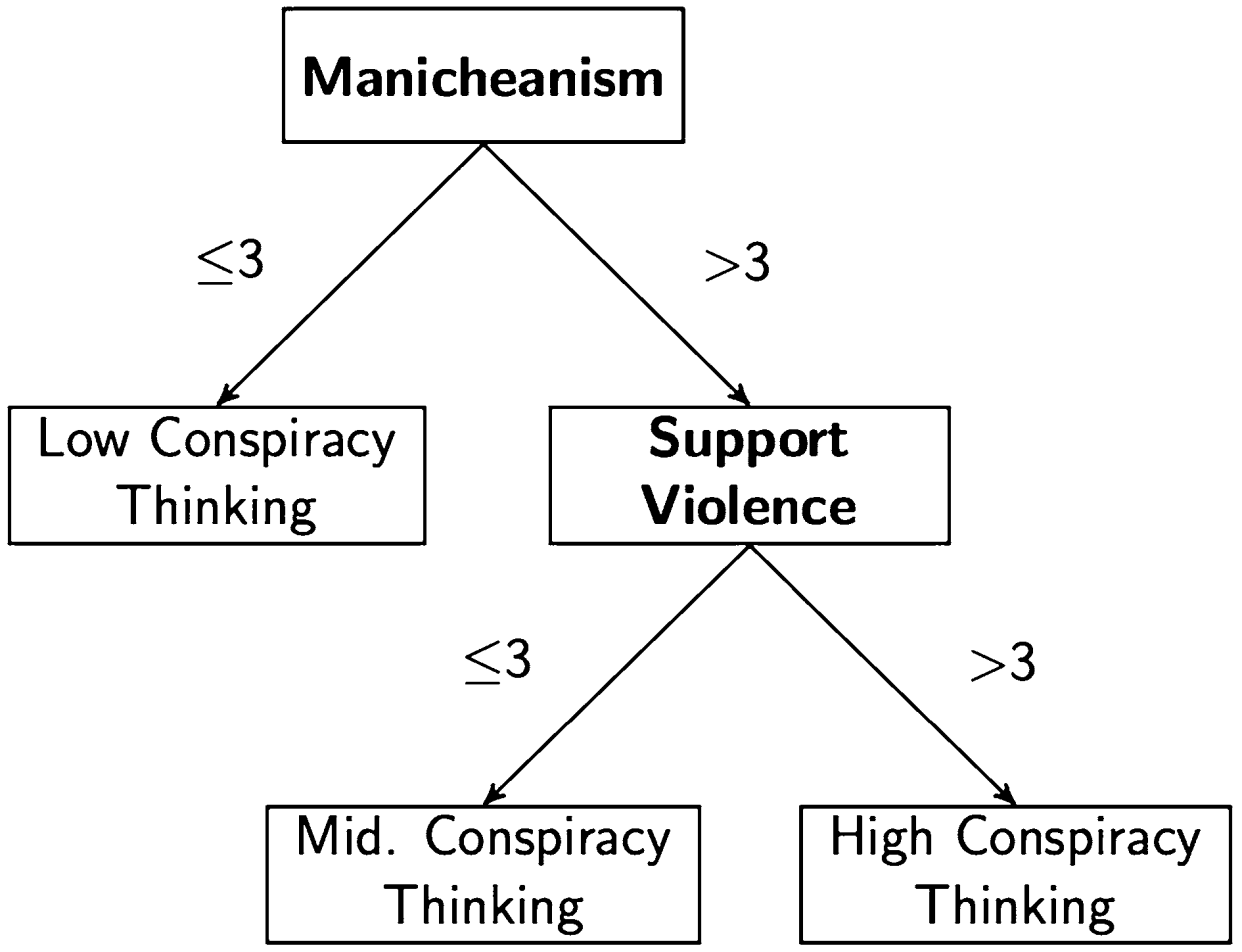
Hypothetical example of a conditional inference tree.

The conditional inference tree procedure begins by searching across the 34 correlates we consider in an attempt to identify the correlate that best organizes the data into two groups. The “best” variable is the one that explains the greatest variance in conspiracy thinking. Imagine, for example, splitting the dataset in two: those above a value of 3 (the midpoint) in Manicheanism, and those at or below a value of 3. If this particular split, on this particular variable—out of all possible combinations of the splits and variables—most accurately classifies those relatively low and high in conspiracy thinking, it is the most predictive (i.e., “best”). This process is recursively applied—conditional on previously determined splits—until no improvements can be made (i.e., until no more variance is explained). For example, we may find that, among those with a value greater than 3 in Manicheanism, additional predictive leverage can be gained by splitting the data between those greater than 3 on support for political violence and everyone else. In this case, those highest in conspiracy thinking would have values greater than 3 on both Manicheanism and support for violence, those lowest in conspiracy thinking would have values of equal to or less than 3 in Manicheanism, and those with a value of Manicheanism greater than 3, but a value equal to or less than 3 in support for violence would be somewhere in between. See the hypothetical example in Fig. 3 for a visualization.

Ultimately, the procedure sequentially selects the variables that best improve the predictive power of the model, and it does not require linear relationships between the predictor variables and conspiracy thinking. Partitioning the data at a particular level of a predictor variable—for example, a 3.5 on the anomie scale—allows for the consideration of the conditional predictive power of variables at differing levels of other predictors. Readers interested in learning more details about these methods may wish to consult other substantive applications^34,35^, including in the conspiracy theory literature^36^. We also recommend consulting the documentation for the programs we use to conduct our analysis, which are described below.

We use a combination of the “party”^28^ and “caret”^37^ R packages to conduct our analysis. First, we randomly partitioned our data into a training sample (75% of observations) and a testing sample (25% of observations). Using the training sample, we estimated the conditional inference tree using the “train()” function, with tenfold cross validation resampling and altering the model tuning parameters. The smallest root mean squared error (RMSE) determined the optimal model. While these decisions comport with best practices to optimize model fit without overfitting, we also note that changes to the granularity of tuning parameters, the resampling method utilized (we also examined the results using bootstrapping and repeated cross-validation), and the number of folds in *K*-fold cross-validation do not alter substantive conclusions. Additional details about the procedure appear in the supplemental information.

## Empirical analysis

### Pairwise correlations

**Figure 4:**
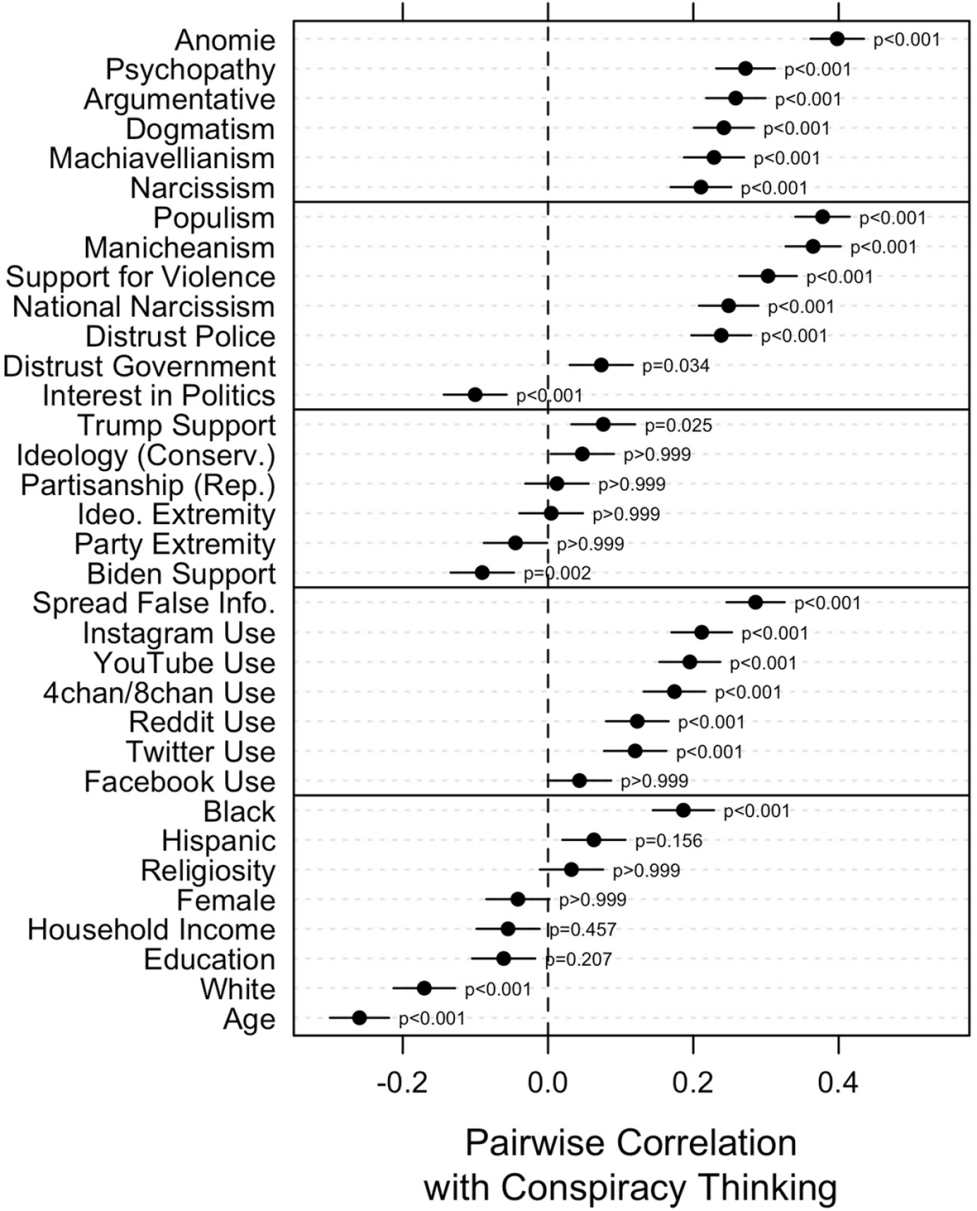
Pearson correlations between ACTS and 34 psychological, political, and social characteristics. Horizontal bars represent 95% confidence intervals. Bonferroni corrections for multiple comparisons were used to compute *p*-values.

We begin our investigation by examining the pairwise correlation between the ACTS and each of the 34 characteristics discussed above, which are presented in Fig. 4. We have organized the correlates in sections as in Table 2. Beginning at the top, we observe consistently strong correlations, ranging from 0.21 to 0.40, between the ACTS and psychological traits. Anomie (e.g., “The situation of the average person is getting worse”), shows the strongest correlation at 0.40 (*p* < 0.001), followed by psychopathy (0.27, *p* < 0.001; e.g., “I tend to be unconcerned with the morality of my actions”), a disposition towards being argumentative (0.26, *p* < 0.001; e.g., “I like to argue online with other people”), dogmatism (0.24, *p* < 0.001; e.g., “It is better to take a stand on an issue even if it’s wrong”), Machiavellianism (0.23, *p* < 0.001; e.g., “I tend to manipulate others to get my way”), and narcissism (0.21, *p* < 0.001; e.g., “I tend to want others to admire me”).

Below these are the non-partisan/ideological political orientations. Within this group of correlates, populism (e.g.., “I would rather be represented by a citizen than by a professional politician”) is the most strongly correlated with conspiracy thinking at 0.38 (*p* < 0.001). This is followed by Manicheanism (0.37, *p* < 0.001; e.g., “Politics is a battle between good and evil”), support for violence (0.30, *p* < 0.001; e.g., “Violence is sometimes an acceptable way for Americans to express their disagreement with the government”), national narcissism (0.25, *p* < 0.001; e.g., “The United States deserves special treatment”), a distrust of police (0.24, *p* < 0.001), distrust of government (0.07, *p* = 0.034), and interest in politics (− 0.10, *p* < 0.001).

We do not observe strong associations between conspiracy thinking and partisan and ideological orientations, or the strength of those orientations. We observe a weak positive association between conspiracy thinking and support for Donald Trump (0.08, *p* = 0.025). Liberal-conservative ideology (0.05, *p* > 0.999) and partisanship (0.01, *p* > 0.999) are not correlated with conspiracy thinking; neither are the extremity of ideological (0.004, *p* > 0.999) or partisan (− 0.04, *p* > 0.999) orientations, regardless of direction. Finally, we observe a weak negative association between conspiracy thinking and support for Joe Biden (− 0.09, *p* = 0.002).

For social media use and activity, we find mostly positive and significant—albeit middling, in magnitude—correlations (0.04–0.29). The strongest association involves one’s propensity to knowingly share false information online (0.29, *p* < 0.001). Time spent on Instagram (0.21, *p* < 0.001) and YouTube (0.20, *p* < 0.001) are more strongly related to conspiracy thinking than time spent on Twitter (0.12, *p* < 0.001) or Reddit (0.12, *p* < 0.001), and the correlation with Facebook usage is not significant (0.04, *p* > 0.999).

Finally, sociodemographic characteristics tend to exhibit weak associations with conspiracy thinking. Identification as Black (0.19, *p* < 0.001) is positively related to conspiracy thinking, though we observe no significant relationship with identification as Hispanic (0.06, *p* = 0.156), gender (− 0.04, *p* > 0.999), religiosity (0.03, *p* > 0.999), educational attainment (− 0.06, *p* = 0.207), or household income (− 0.05, *p* = 0.457). Age (− 0.26, *p* < 0.001) and identification as White (− 0.17, *p* < 0.001) are significantly negatively related to conspiracy thinking.

### Conditional inference tree

Figure 4 tells us about the strength of the bivariate relationships between conspiracy thinking and various correlates which have been previously identified in the literature. But our goal is to understand which factors seem to be most informational relative to the others. When pitted against each other, which correlates seem to best account for variability in conspiracy thinking? To answer this question, we turn to the conditional inference tree analysis.

**Figure 5:**
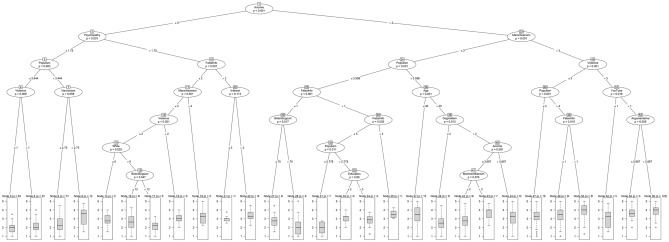
Conditional inference tree results. Nodes (in circles) represent predictor variables. At the bottom of each branch is the distribution of the ACTS among individuals in that branch (i.e., with the associated profile of characteristics), where 5 represents the highest level of conspiracy thinking. Bonferroni corrections for multiple comparisons were used to compute *p*-values.

First, we note that the predictive power of our model is weak-moderate. We used the model depicted in Fig. 5—which was estimated on our training sample—to predict the ACTS in the testing sample that we reserved. The squared correlation between the model-based predictions and the data is 0.24.

The central output of the conditional inference tree analysis is the tree that appears in Fig. 5. At the bottom of the tree are 28 different box plots displaying the distribution of conspiracy thinking (which ranges from 1 to 5). Tracing up from each box plot, we can see the characteristics of individuals displayed in that branch of the tree. Consider, for example, the box plot that is farthest to the right of the figure, which displays a distribution heavily skewed toward the conspiratorial end of the ACTS. Tracing the branches (lines) upward, we notice that five nodes—the ovals which correspond to predictor variables—are involved (from bottom to top): argumentative, YouTube, violence, Manicheanism, and anomie. More specifically, as can be determined by the numerical values appearing in the center of each branch, those individuals high in conspiracy thinking have values greater than 2.667 on the argumentative scale (i.e., neutral or more argumentative), greater than 1 on YouTube use (once a month or greater), and greater than 3 (the neutral position) when it comes to support for political violence, Manicheanism, and anomie.

Next, consider the box plot on the far-left side of the tree, which depicts individuals that are generally low on the ACTS. The individuals classified here show values equal to or less than 3 (neutral) on anomie, less than or equal to 1.75 on psychopathy (disagreeing with items, on average), less than or equal to 3.444 on populism (neutral or non-populist), and less than or equal to 1 when it comes to support for political violence (strongly disagreeing with violence, on average). In addition to the variables already mentioned, the propensity to share false information online, dogmatism, narcissism, psychopathy, Machiavellianism, interest in politics, Biden support, age, educational attainment, and self-identification as White are all useful in classifying individuals along the conspiracy thinking continuum—16 distinct predictors of the original 34 considered.

The variables that appear to be the most useful in orienting individuals along the ACTS scale are psychological; indeed, all of the psychological traits we consider appear in the final tree. Second most important are non-partisan/ideological political orientations, including populism, Manicheanism, support for political violence, and interest in politics. Note, however, that (dis)trust in government, (dis)trust in police, and national narcissism do not appear in the tree. When it comes to partisan/ideological political orientations only support (or lack thereof) for Joe Biden is useful; mainstream partisan and ideological identities are not particularly useful in distinguishing levels of conspiracy thinking. Two variables dealing with social media activity and behavior are important: the propensity to share false information online and YouTube use. The former appears in the tree many times, appearing particularly helpful in distinguishing between middling levels of conspiracy thinking. Notably, time spent on Facebook, Twitter, Instagram, and Reddit are not particularly useful in explaining conspiracy thinking. Finally, age, educational attainment, and White identification also appear to be useful in distinguishing between middling levels of conspiracy thinking, though religiosity, gender, income, and identification as Black or Hispanic do not.

To explore this information in a different way, we present the average profile of individuals in the first and third terciles of the ACTS along each of the 16 variables identified by the conditional inference tree analysis in Table 3; we also include the grand sample means of each correlate for reference. These two individuals are both likely to be White, middle-aged, and possessing of middling levels of educational attainment. They also tend to frequent YouTube and be fairly interested in politics.

**Table 3:** Average profile of respondents exhibiting low and high levels of conspiracy thinking across the variables identified by the conditional inference tree analysis. Numerical values represent scores on the variables listed in the first column.

Variable	Low ACTS	High ACTS	Grand mean
Anomie	3.25	3.94	3.56
Argumentative	2.59	3.23	2.86
Dogmatism	2.92	3.41	3.13
Machiavellianism	1.87	2.30	2.11
Narcissism	2.11	2.59	2.35
Psychopathy	1.88	2.36	2.13
Interest in politics	4.27	4.10	4.12
Manicheanism	2.80	3.74	3.27
Populism	3.61	4.17	3.84
Support political violence	1.78	2.62	2.20
Biden support	53.49	46.94	49.51
YouTube usage	3.39	4.01	3.73
Share false info. online	1.43	2.16	1.81
Age	49.81	40.38	44.67
Education	3.78	3.67	3.69
White*	0.77	0.61	0.68

*Proportion rather than mean.

As for the other characteristics, inferences are more complicated. Individuals registering high on the ACTS do, as the conditional inference analysis above shows, exhibit higher levels of anomie, predispositions toward argumentation and sharing false information online, dogmatism, Machiavellianism, psychopathy, Manicheanism, populism, and support for political violence than the individual low in ACTS. Moreover, most of the values for the high ACTS individual are also greater than the average across the full sample; in other words, they are greater than the average American, as well as those low in conspiracy thinking. However, it is also worth noting that, while high ACTS individuals are *relatively* high in these psychological and political characteristics, they are not always high *in the absolute*. Exceptions may include anomie, Manicheanism, and populism, for which the average score corresponds to an average response of “agree” across the items that compose each scale for those high on the ACTS. Many of the remaining characteristics—e.g., narcissism, psychopathy—are more likely, but not likely in the absolute, for high ACTS individuals compared to low ACTS individuals. Hence, researchers should be circumspect regarding the inferences they make about who conspiracy theorists are and take seriously the fact that prediction of conspiracy thinking—even using a large number of previously-identified correlates spanning the psychological, political, and social domains—remains elusive, on balance.

## Discussion

There are a seeming infinite number of conspiracy theories, and infinite versions thereof^38^. Beliefs in individual conspiracy theories are often related to different factors^6^, leaving researchers unable to accurately generalize about the correlates of conspiracy theory beliefs. Moreover, the salience, political consequences, and, therefore, correlates of specific conspiracy theories change over time, further complicating inference^30^. But if we understand the underlying psychology of conspiracism (and the contexts that support or constrain it), researchers may be able to overcome the problems that hinder generalizable inference. For this and other reasons, researchers have theorized and measured a general predisposition toward conspiracism^18–20^, which has been found to correlate with beliefs in numerous conspiracy theories^24^. This predisposition, or tendency, to interpret events and circumstances as the product of conspiracies has not, however, been comprehensively investigated by researchers, leaving open even basic questions about the correlates of conspiracy thinking.

In this study, we examined 34 predictors from a variety of substantive domains. First, we found that many of the previously identified correlates of specific conspiracy theory beliefs are related to conspiracy thinking as well. Second, and most importantly, we deciphered which correlates were the most useful in explaining variance in conspiracy thinking. We found that psychological factors—particularly anti-social personality traits, such as the dark triad, dogmatism, and a tendency to be argumentative—and non-partisan/ideological political orientations—Manicheanism, populism, support for political violence—were strong, significant predictors, whereas partisan and ideological orientations, social media activity, and sociodemographic characteristics are generally less predictive, at least when other characteristics are accounted for.

Despite the number and variety of hypothesized predictors included here, conspiracy thinking is difficult to accurately predict. On the one hand, new and better measures of potential correlates of conspiracy thinking could, over time, change this result. As the still nascent literature develops, researchers will become better equipped to account for variability in conspiracy thinking. On the other hand, there may be causal antecedents of conspiracy thinking—socialization, genetics, evolutionary factors, idiosyncratic situational factors and experiences—that ensure the predisposition will always be difficult to predict with a high degree of accuracy, as is the case for a great many social-psychological constructs.

Even though accurately predicting how conspiratorial one is appears to be a difficult task that the literature is not (yet) equipped to accomplish with a great deal of accuracy, we do possess evidence that high levels of anomie, Manicheanism, support for political violence, populism, and the propensity to argue online are related to the highest levels of conspiracy thinking. These individuals are relatively pessimistic (e.g., anomie) about a world controlled by corrupt elites (e.g., populism) who they see in terms of good and evil (e.g., Manicheanism), and they are willing to behave in nonnormative ways to serve their own ends (e.g., supporting violence against the government, arguing online). These are disconcerting traits that social scientists should be more diligent about probing and understanding; these are also traits that social and political leaders should work to address. As recent events, such as the “Pizzagate” event and the January 6, 2021 attack on the U.S. Capitol, suggest, even a small number of individuals exhibiting behavior consistent with these types of anti-social personality traits and conflictual interpersonal styles can impact the greater, less extreme public^39^.

The patterns we observe bode poorly for efforts at correcting or minimizing the impact of conspiracy theory beliefs, at least as they are typically deployed at this point in time: those who exhibit the highest levels of conspiracy thinking also possess psychological and political traits (e.g., dogmatism, argumentative, distrusting, narcissistic) that would seemingly make them hostile to perceived corrections or interventions from outsiders. Our evidence should be used to inform the development of future interventions. Current approaches at corrective measures might show low or inconsistent efficacy precisely because of some of the psychological tendencies identified here. But a greater understanding of how conspiratorial thinking is socialized and supported by peers could inform different approaches (e.g., inoculation methods to prevent persuasion^40–45^; subtle shifts in norms or message accessibility^46,47^; the removal of bad actors and online bots^48^). Epidemiological susceptible–infected–removed (SIR) models, which have been successfully applied to the online spread of rumors, may pose a fruitful avenue for addressing conspiracy theory beliefs because they take more seriously than most pre-bunking and correction methods the characteristics of those who are susceptible to spreading rumors and most likely to be “infected”^49^. In other words, our findings could be used to calibrate SIR models applied to the spread of conspiracy theories.

Our results also highlight the differences between beliefs in specific conspiracy theories and the general disposition towards seeing events and circumstances in conspiratorial terms^16^. For example, partisanship and liberal-conservative ideology are frequently associated with beliefs in specific conspiracy theories, such as those about climate change^13,50,51^, Barack Obama’s birth certificate^17,52^, COVID-19^12^, and voter fraud^8,53,54^, suggesting that if a conspiracy theory has partisan or ideological content, or is proffered by partisan elites on one side, it will likely be believed more by one partisan/ideological group than by others. However, our model does not find partisanship, liberal-conservative ideology, or the extremity of either predisposition to be uniquely predictive of generalized conspiracy thinking. This finding contributes to the ongoing debate in which studies variously find that conspiracy thinking is more prominent among the political right^13^, among extremists of left *and* right^27^, among independents^55^, or nearly even between left and right^20^. Our findings align most closely with the latter studies, we surmise, because our analysis includes numerous psychological and non-partisan/ideological predictor variables that have not been included in previous studies, which tend to focus on bivariate relationships. That said, our findings do not preclude partisanship and liberal-conservative ideology from, at times, being more strongly related to conspiracy thinking. As emerging research indicates, partisan elites can use conspiratorial rhetoric to activate conspiracism and subsequently mobilize conspiracy-minded people to action for partisan ends^56^. This view is consistent with the correlations in Fig. 4, which show, as we might expect given Donald Trump’s frequent use of conspiratorial rhetoric, that Trump support is positively correlated with conspiracy thinking, while support for Biden, who generally refrains from employing such rhetoric, is negatively correlated.

Our investigation is not without limitations. First, even though the methods we employ are designed to aid in prediction, they are not causal; therefore, the caveats that usually accompany observational data apply. It could be the case that conspiracy thinking causes the characteristics we examine above, rather than the opposite. Substantively, both causal directions could make sense: for example, believing powerful groups are conspiring could lead one to be pessimistic about the future (e.g., anomie), but anomie could also prime one to use conspiracy theories to explain society’s presumed decline.

Second, we could not include every correlate of conspiracy theory beliefs previously identified in the rapidly expanding literature. That said, we suspect that an even longer list of correlates would demonstrate the general importance of anti-social and non-mainstream psychological traits and political orientations, as recent research is increasingly finding^6,56^. Likewise, while more correlates would surely increase the predictive power of our model, we are skeptical that simply adding a few additional social-psychological constructs would alter our general conclusion that conspiracy thinking is, at this stage in the research program, difficult to predict with precision. Relatedly, we also acknowledge that there are a handful of viable scales for measuring conspiracy thinking^18,19^ that could also be used to expand and replicate the analysis presented here.

Finally, we recognize that our analyses were conducted on a U.S. sample at a relatively contentious time in American politics (i.e., just before an election during a pandemic). Therefore, we urge future studies to expand and replicate this analysis in other sociopolitical and situational contexts and encourage efforts to examine conspiracism in a comparative tradition^27,57^.

## Supplementary Information

Supplementary Information.

## Data Availability

Further details about the data and analysis are available in the supplementary information files. All data and replication code are available via the Open Science Framework: https://osf.io/t28b4/.
