# Supplementary material for: On modeling the correlates of conspiracy thinking
Source: Sci Rep. 2023 May 23;13:8325. doi: 10.1038/s41598-023-34391-6 (PMC10204035; doi:10.1038/s41598-023-34391-6)
Supplement: Supplementary file 1 — Supplementary Information. [file 41598_2023_34391_MOESM1_ESM.pdf]

Supplementary Information

“On Modeling the Correlates of Conspiracy Thinking”

Table of Contents

I. Question wording and variable coding, pages 2–5

II. Conspiracy theory belief questions, page 6

III. Sample composition and survey details, page 7

IV. Correlations between predictors, page 8

V. Variable importance, page 9

VI. Details about conditional inference tree analysis, page 10

VII. Results from linear regression analysis, page 11

NOTE: All data and replication files can be found on the Open Science Framework: https://osf.io/t28b4/.

I. Question wording and variable coding

Anomie. Please tell us how much you agree or disagree with each of the statements below (each item is 1=strongly disagree, 5=strongly agree):

1. The situation of the average person is getting worse.
2. It is hardly fair to bring a child into today's world.
3. Most public officials are not interested in the average person.

Populism. Please tell us how much you agree or disagree with each of the statements below (each item is 1=strongly disagree, 5=strongly agree):

1. Politicians always end up agreeing when it comes to protecting their privileges.
2. Politicians should follow only the will of the people.
3. The people, and not politicians, should make our most important policy decisions.
4. The political differences between the elite and the people are much larger than the differences among the people.
5. I would rather be represented by a citizen than by a professional politician.
6. Elected officials talk too much and take too little action.
7. What people call "compromise" in politics is really just selling out on one's principles.
8. Established politicians who claim to defend our interests, only take care of themselves.
9. The established elite and politicians have often betrayed the people.

Distrust Police. Please tell us how much you agree or disagree with each of the statements below: "I trust the police."

1. Strongly agree
2. Agree
3. Neither agree nor disagree
4. Disagree
5. Strongly disagree

Distrust Government. Please tell us how much you agree or disagree with each of the statements below: "The federal government in Washington can be trusted to do what is right."

1. Strongly agree
2. Agree
3. Neither agree nor disagree
4. Disagree
5. Strongly disagree

Machiavellianism. Please tell us how much you agree or disagree with each of the statements below (each item is 1=strongly disagree, 5=strongly agree):

1. I tend to manipulate others to get my way.
2. I have used deceit or lied to get my way.
3. I have used flattery to get my way.
4. I tend to exploit others towards my own end.

Narcissism. Please tell us how much you agree or disagree with each of the statements below (each item is 1=strongly disagree, 5=strongly agree):

1. I tend to want others to admire me.
2. I tend to want others to pay attention to me.
3. I tend to seek prestige or status.
4. I tend to expect special favors from others.

Psychopathy. Please tell us how much you agree or disagree with each of the statements below (each item is 1=strongly disagree, 5=strongly agree):

1. I tend to lack remorse.
2. I tend to be unconcerned with the morality of my actions.
3. I tend to be callous or insensitive.
4. I tend to be cynical.

Argumentative. Please tell us how much you agree or disagree with each of the statements below (each item is 1=strongly disagree, 5=strongly agree):

1. I like to argue online with other people.
2. I enjoy a good argument over a controversial issue.
3. I am willing to express my opinion online even if others strongly disagree with me.

Share false information. I share information on social media about politics even though I believe it may be false.

1. Strongly disagree
2. Disagree
3. Neither agree nor disagree
4. Agree
5. Strongly agree

Social media use. Thinking about the social media you use, how often in a typical week do you visit or use: 1) Facebook, 2) Twitter, 3) Instagram, 4) Reddit, 5) 4chan, 8chan, 6) YouTube:

1. Not at all
2. Once a month or less
3. Several times a month
4. Several times a week
5. Everyday

Dogmatism. Please tell us how much you agree or disagree with each of the statements below (each item is 1=strongly disagree, 5=strongly agree):

1. On important public issues, I believe you should either be for them or against them and not take a middle course.
2. It is better to take a stand on an issue even if it's wrong
3. When it comes to the really important questions about religion and philosophy of life, a person must decide them, one way or the other.

National narcissism. Please tell us how much you agree or disagree with each of the statements below (each item is 1=strongly disagree, 5=strongly agree):

1. The United States deserves special treatment.
2. Not many people seem to fully understand the importance of the United States.
3. I will never be satisfied until the United States gets the recognition it deserves.

Manicheanism. Politics is a battle between good and evil.

1. Strongly disagree
2. Disagree
3. Neither agree nor disagree
4. Agree
5. Strongly agree

Support violence. Violence is sometimes an acceptable way for Americans to express their disagreement with the government.

1. Strongly disagree
2. Disagree
3. Neither agree nor disagree
4. Agree
5. Strongly agree

Conspiracy thinking. (each item is 1=strongly disagree, 5=strongly agree):

1. Much of our lives are being controlled by plots hatched in secret places.
2. Even though we live in a democracy, a few people will always run things anyway.
3. The people who really 'run' the country, are not known to the voters.
4. Big events like wars, the current recession, and the outcomes of elections are controlled by small groups of people who are working in secret against the rest of us.

Interest in politics. "Some people follow what's going on in politics and current events most of the time. Others aren't that interested. How often do you follow what's going on in government and current events?"

1. Never
2. Hardly at all
3. Only now and then
4. Some of the time
5. Most of the time

Ideology. Self-placement; 1=extremely liberal, 7=extremely conservative.

Partisanship. Self-placement; 1=strong Democrat, 7=strong Republican.

Party strength. "Folded" partisanship measure.

1. True independent
2. Lean toward party
3. Weak partisan
4. Strong partisan

Ideological strength. “Folded” ideology measure.

1. Moderate/centrist
2. Leaner
3. Weak identifier
4. Strong identifier

Trump support. “Feeling thermometer” ranging from 0 (very “cold,” negative) to 100 (very “warm,” positive).

Biden support. “Feeling thermometer” ranging from 0 (very “cold,” negative) to 100 (very “warm,” positive).

Religiosity. On average, I attend religious services or a place of worship: (please do not include weddings or funerals

1. Never
2. A few times a year
3. Once or twice a month
4. Once a week
5. Every day

Sociodemographics:

1. Educational attainment (6-point scale, 1=No high school degree, 5=post-grad degree)
2. Age (age in years, 18–90)
3. Household income (7-point scale, 1=$24,999 or less, 7=200,000 or more)
4. Gender (0=male, 1=female)
5. Race (Black: 0=not Black, 1=Black; Hispanic: 0=not Hispanic, 1=Hispanic)

II. Conspiracy theory belief questions

Table A1: Percentage of respondents who "agree" or "strongly agree" with each item.

Label	Conspiracy Theory Belief Question	% Agree
Epstein Death	Jeffrey Epstein, the billionaire accused of running an elite sex trafficking ring, was murdered to cover-up the activities of his criminal network.	52
Deep State	There is a "deep state" embedded in the government that operates in secret and without oversight.	41
Post Office Fraud	There is a conspiracy to stop the U.S. Post Office from processing mail-in ballots.	41
Elite Trafficking	Elites, from government and Hollywood, are engaged in a massive child sex trafficking racket.	35
COVID Exaggerated	The threat of coronavirus has been exaggerated by political groups who want to damage President Trump.	31
Anti-vaxx	The dangers of vaccines are being hidden by the medical establishment.	28
5G Cover-up	The dangers of 5G cellphone technology are being covered up.	23
Climate Hoax	Climate change is a hoax perpetrated by corrupt scientists and politicians.	18
False Flag	School shootings, like those at Sandy Hook, CT and Parkland, FL are false flag attacks perpetrated by the government.	12
QAnon Believer	I am a believer in QAnon.	7

III. Sample composition and survey details

Table A2: Sociodemographic information about October 2020 sample, compared to 2010 U.S. Census estimates.

Characteristic	October 2020	2010 Census Estimate
Age (median)	43	38
High school degree (%)	97	88
Some college or more (%)	76	59
Female (%)	51	51
Household income (median)	$25,000–$49,999	$49,445
Race:		
White (%)	68	72
Black (%)	14	13
Hispanic (%)	17	16
n	2,015	

Note: All entries are percentages except age, which is the median.

While median age is slightly higher for our survey than the 2010 Census estimates, recall that we are able to poll on only those age 18 years old and older, whereas the Census provides the median age of all Americans.

IV. Correlations between predictors

Figure A1: Pearson correlations between predictor variables.

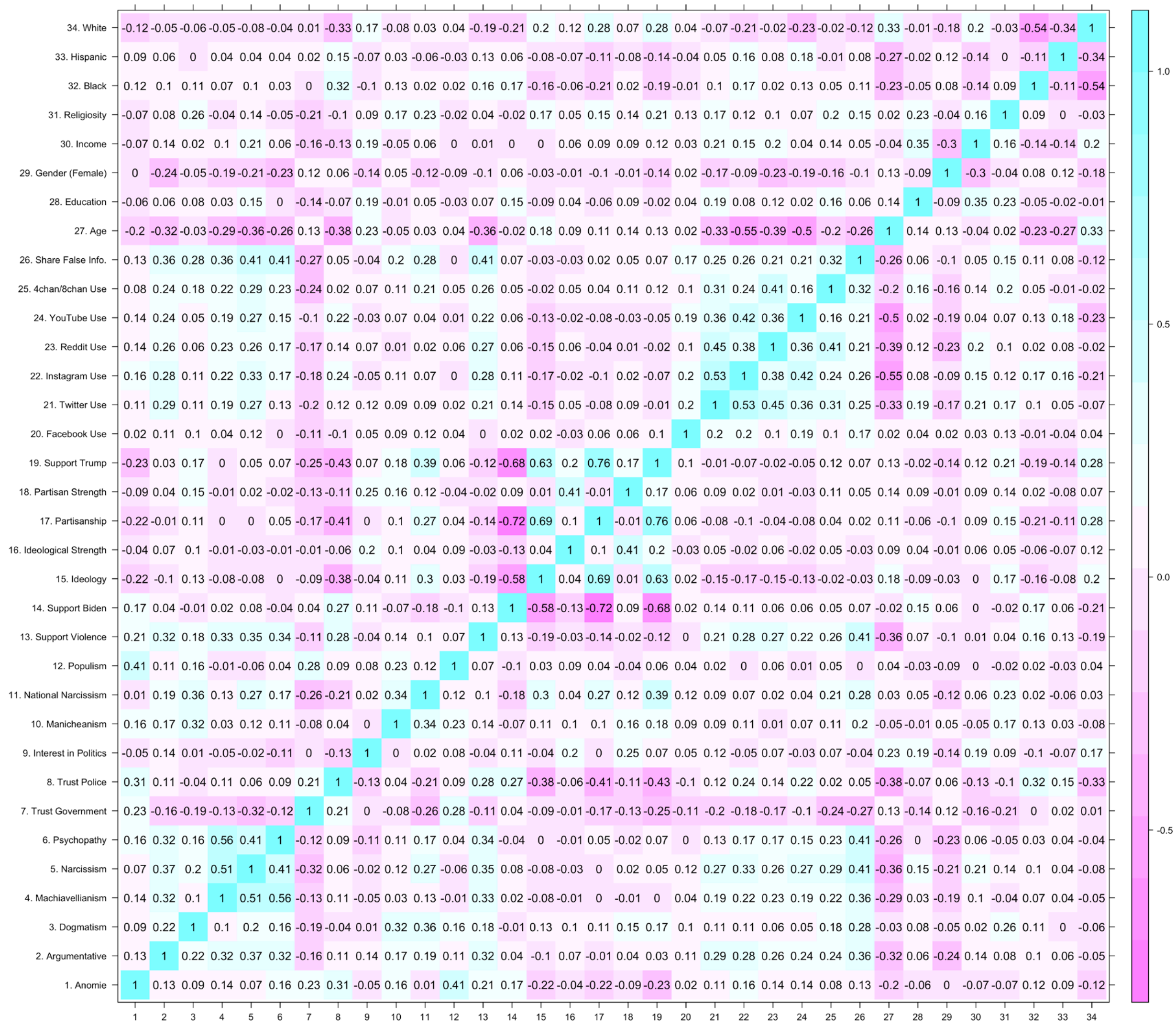

V. Variable importance

Because the correlations presented in Figure 3 assume linear relationships between conspiracy thinking and each predictor, we also examined the strength of the relationship as operationalized by a LOESS (locally-weighted non-parametric) regression of the former on the latter. The following figure ranks the predictors based on the R^2 from the LOESS. Comparing Figure A1 to Figure 3, substantive patterns appear to be quite similar, suggestions that deviations from linearity are generally minimal.

The "varImp()" function in the "caret" R package was used to execute this analysis and produce the figure.

Figure A2: Variable importance as determined by the R^2 value from a LOESS (locally weighted) regression of the ACTS on each correlate.

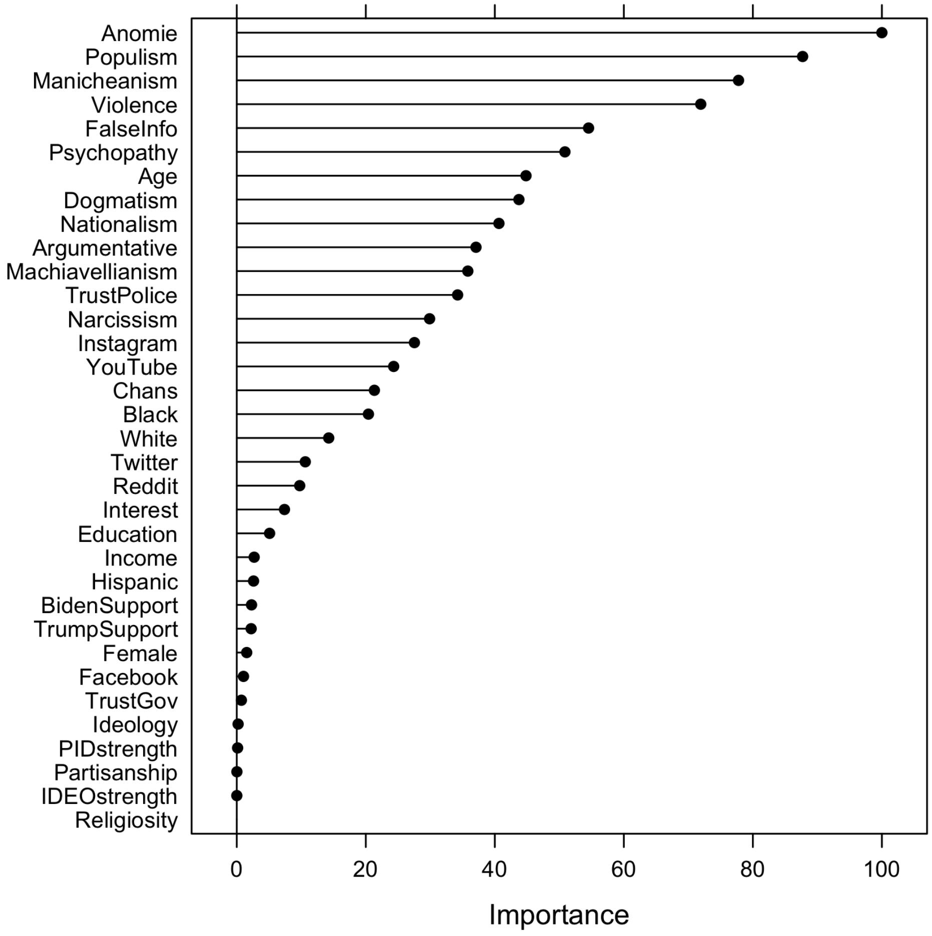

VI. Details about conditional inference tree analysis

We use a combination of the "party" and "caret" R packages to conduct our analysis. First, we randomly partitioned our data into a training sample (75% of observations) and a testing sample (25% of observations). Using the training sample, we estimated the conditional inference tree using the "train()" function, with 10-fold cross validation resampling and altering the model tuning parameters—1-*p*-value threshold, called "mincriterion"—10 times. The model/tuning parameter with the smallest root mean squared error (or largest R^2) determined the optimal model.

Figure A3: Change in Root Mean Squared Error (RMSE) across the tuning parameter, 1–*p*-value threshold ("mincriterion").

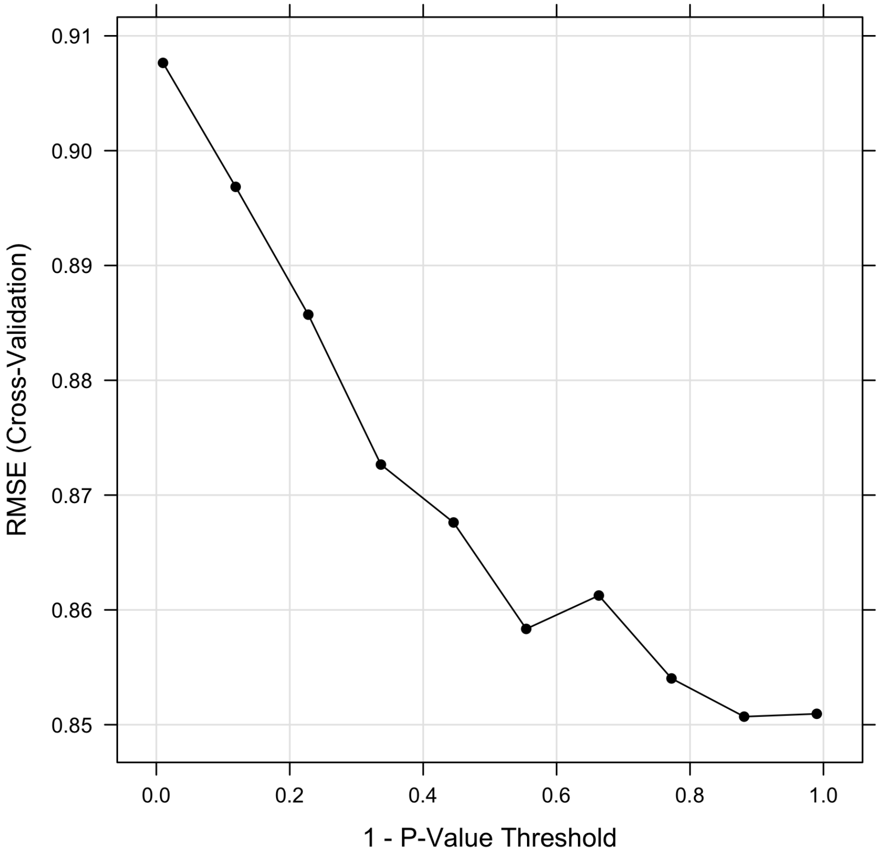

VII. Results from linear regression analysis

Below we present the results of an OLS regression of the ACTS on all 34 predictors.

Table A3: Results from linear regression of ACTS on 34 correlates.

	Coefficient	S.E.	p-value
Anomie	0.223^{***}	0.026	0.000
Argumentative	0.047^{*}	0.021	0.023
Dogmatism	0.041	0.024	0.085
Machiavellianism	0.056^{*}	0.024	0.020
Narcissism	0.024	0.023	0.291
Psychopathy	0.055^{*}	0.026	0.033
Populism	0.305^{***}	0.032	0.000
Manicheanism	0.139^{***}	0.017	0.000
Support Violence	0.055^{***}	0.016	0.001
National Narcissism	0.087^{***}	0.022	0.000
Distrust Government	0.067^{***}	0.019	0.000
Distrust Police	0.073^{***}	0.018	0.000
Interest in Politics	-0.041^{*}	0.020	0.040
Trump Support	0.002^{**}	0.001	0.002
Biden Support	-0.003^{***}	0.001	0.000
Partisanship (Rep.)	-0.051^{***}	0.014	0.000
Ideology (Conserv.)	0.047^{***}	0.014	0.001
Party Extremity	-0.024	0.018	0.196
Ideological Extremity	-0.014	0.017	0.387
Share False Info.	0.075^{***}	0.020	0.000
YouTube Use	0.027	0.014	0.065
4chan/8chan Use	0.053	0.027	0.051
Instagram Use	0.021	0.013	0.103
Reddit Use	-0.003	0.016	0.856
Twitter Use	-0.014	0.013	0.291
Facebook Use	-0.008	0.012	0.472
Religiosity	-0.018	0.015	0.236
Age	-0.002	0.001	0.184
Education	-0.004	0.014	0.772
Female	0.045	0.038	0.245
Household Income	-0.005	0.011	0.689
Black	0.116	0.066	0.078
Hispanic	-0.031	0.053	0.555
White	-0.094	0.052	0.069
Constant	-0.475^{*}	0.207	0.022
R^2	0.423		
n	1,949		

Note: OLS coefficients. * $p < 0.05$, ** $p < 0.01$, *** $p < 0.001$
